# The Role of Buffers in Wild-Type HEWL Amyloid Fibril Formation Mechanism

**DOI:** 10.3390/biom9020065

**Published:** 2019-02-14

**Authors:** Sandi Brudar, Barbara Hribar-Lee

**Affiliations:** Faculty of Chemistry and Chemical Technology, University of Ljubljana, Večna pot 113, SI-1000 Ljubljana, Slovenia; sandi.brudar@fkkt.uni-lj.si

**Keywords:** lysozyme, amyloid fibrils, buffer-specific effects

## Abstract

Amyloid fibrils, highly ordered protein aggregates, play an important role in the onset of several neurological disorders. Many studies have assessed amyloid fibril formation under specific solution conditions, but they all lack an important phenomena in biological solutions—buffer specific effects. We have focused on the formation of hen egg-white lysozyme (HEWL) fibrils in aqueous solutions of different buffers in both acidic and basic pH range. By means of UV-Vis spectroscopy, fluorescence measurements and CD spectroscopy, we have managed to show that fibrillization of HEWL is affected by buffer identity (glycine, TRIS, phosphate, KCl-HCl, cacodylate, HEPES, acetate), solution pH, sample incubation (agitated vs. static) and added excipients (NaCl and PEG). HEWL only forms amyloid fibrils at pH = 2.0 under agitated conditions in glycine and KCl-HCl buffers of high enough ionic strength. Phosphate buffer on the other hand stabilizes the HEWL molecules. Similar stabilization effect was achieved by addition of PEG12000 molecules to the solution.

## 1. Introduction

In the recent years an increased body of evidence came to existence, connecting the formation of amyloid fibrils, highly ordered protein aggregates, to several neurological diseases [1]; so far, about 23 unrelated proteins have been found to be amyloidogenic, and associated with clinically distinct conditions [2] (A review of the neurodegenerative diseases is given [3]). Since the global population is still growing and almost every country in the world is facing a substantial increase in proportion of seniors in their population, these neurodegenerative disorders that are often age-related present a growing threat to mankind. Understanding the physicochemical forces that drive such aggregation and its mechanism is crucial for development of drugs for these diseases, so it is of no surprise that the process of these pathognomonic structure formation has been, and still is, a subject of numerous studies. In spite all these efforts, there are still many fundamental physical and chemical questions about the molecular mechanism of amyloid fibril formation that remain unanswered [4].

Lysozyme is a protein whose structure, stability, and folding have been extensively studied under various conditions. Human lysozyme upon various mutations has been shown to cause systemic nonneuropathic amyloidosis [2,5]. Hen egg-white lysozyme (HEWL) structural and functional features are very similar to those of human lysozyme (around 60% sequence similarity [6]), and the protein is at the same time easily available which makes it a good model protein to investigate amyloidogenesis [2]. Further, significant amount of data exist on various conditions that successfully induce/inhibit HEWL in vitro fibrillization (for review see [2,5,6]). It’s been established that, as in general all intermolecular interactions, the process of amyloid fibrillization depends on two factors, the intrinsic properties of the protein molecule, and on the environment in which the protein is situated. Since the amyloid aggregates are characterized by a cross-beta sheet quaternary structure [7,8,9] the protein needs to undergo partial denaturation before forming these aggregates. For HEWL “in vitro” this can be triggered by change in pH, change in temperature, or addition of different co-solutes, such as simple salts, ethanol, guanidine hydrochloride, urea, and other destabilizing agents into the solution [2,5,10]. Ions, for example, depending on their characteristics, tend to accumulate or deplete from the solute/water interface [11,12], which is accompanied by hydration/dehydration process. They further influence the $pK_{a}$ values of the protein amino acids, influencing the protein net charge [13]. Guanidine hydrochloride, on the other hand, does not influence the solvation of the protein directly but due to its polarization changes the electrostatic potential in its surface [14]. For alcohols in general, the suggested mechanism of denaturation is by weakening hydrophobic bonds through clathrate structure interruption [15]. To sum up, all the chemical components present in the protein environment would influence the protein stability.

The studies of protein stability are usually carried out in vitro, where the conditions are distinctively different from those in vivo, but are at the same time much simpler and therefore give us valuable information about protein interactions. Never-the-less, even in vitro the proteins are not the only components in the solutions. To maintain the protein native structure the use of buffer solutions is indispensable. It’s been established in the last couple of years that the buffer does not merely maintain the appropriate pH of the solution at which the protein is stable in its native structure but can actually influence the stability of the protein itself. If the buffer molecules preferentially bind to the native state of the protein, this would stabilize the protein, while in the cases where buffer molecules bind selectively to the denatured state, this would lead to destabilization of the protein native structure [16,17,18]. Buffers also influence the hydration shell of protein molecules which has an impact on the protein stability. They would influence thermally induced aggregation of proteins [19,20], stability of the antibody formulations [21], electrophoretic mobility of proteins [22], to mention just a few. Buffers further modulate ion specific effects, that are known to have a strong influence on the protein stability, as well as on the protein-protein interactions [23,24,25,26,27,28].

Even though all this experimental evidence exists about their importance, the role of buffers in the studies of amyloid fibril formation has not yet been systematically investigated, which could be one of the reasons that pieces of information regarding the process are sometimes contradictory. In this work, we systematically explored the influence of the buffer used on the formation of amyloid fibrils in the solutions of HEWL. Different buffers (glycine, tris(hydroxymethyl)aminomethane (TRIS), cacodylate, KCl-HCl, acetate, phosphate, and 2-[4-(2-hydroxyethyl)piperazin-1-yl]ethanesulfonic acid (HEPES)) were used at different ionic strengths, as well as at different pHs. The results were interpreted in the view of buffer specific effects.

## 2. Materials and Methods

### 2.1. Materials

HEWL, glycine, sodium dihydrogen phosphate dihydrate, sodium chloride, acetic acid and hydrochloric acid were purchased from Merck (Darmstadt, Germany). Thioflavin T, Congo red, poly(ethylene glycol)12000 ($M_{w}=12,000$ g/mol) (PEG12000), HEPES, cacodylic acid and TRIS were purchased from Sigma-Aldrich (St. Louis, MI, USA). Di-Sodium hydrogen phosphate was purchased from Chem-Lab (Zedelgem, Belgium) and 8-anilinonaphthalene-1-sulfonic acid ammonium salt (8,1-ANS $NH_{4}$-salt) from Fluka-Biochemika (Buchs, Switzerland).

### 2.2. Buffers

In order to evaluate buffer-specific effects in HEWL solutions, we have prepared several buffers with different ionic strength. The buffers were filtered through 0.45 μm filter pores (Sartorious) before use.

### 2.3. Fibril Preparation

We prepared 14.3 mg/mL HEWL solutions in various buffers. HEWL solutions were then incubated at 37 °C under agitated or static conditions. Agitation was performed in an orbital incubator (Sanyo) at 150 rpm for 7 days. Protein samples under static conditions were kept in a thermostat for 15 days (Huber). Fresh HEWL solutions in corresponding buffers were prepared on the day of agitated sample analysis.

### 2.4. UV Measurements

Congo red dye binding assay was used for detection of amyloid fibrils. Its aqueous solutions have an absorption maximum at 490 nm. Binding of Congo red to amyloid fibrils induces a red shift in its maximum absorbance wavelength from 490 to 540 nm and/or leads to an increase in sample absorbance [29]. 10 μM aqueous Congo red solution was mixed with 20 μM fresh, agitated and static HEWL solutions at 1:1 (Congo red:protein) ratio. After subsequent incubation of mixtures for 30 min at room temperature, absorbance spectra were recorded from 700 to 360 nm.

### 2.5. Fluorescence Quenching

Although cases have been reported where fluorescent dye thioflavin T (ThT) can yield to false negative results [30], ThT has become one of the most established “gold standards” for amyloid detection both in vitro and in vivo. Upon binding to amyloid aggregates its excitation maximum moves from 385 nm to 450 nm and its emission intensity amplifies at 485 nm [31]. 100 μM HEWL samples were incubated for 5 min with 50 μM ThT. After excitation at 450 nm fluorescence emission spectra were measured from 460 nm to 560 nm to investigate the amyloid nature of HEWL solutions. 1-anilinonaphthalene-8-sulfonate (ANS) dye is used to ascertain the extent of exposed hydrophobic regions on protein surface. ANS preferentially binds with them, which increases its fluorescence intensity at 480 nm [32]. Fluorescence emission spectra of solutions containing 50 μM HEWL and 200 μM ANS were recorded from 400 nm to 600 nm after excitation at 380 nm. For both ThT and ANS measurements corresponding baseline curves were subtracted from raw data.

### 2.6. Circular Dichroism (CD)

Amyloid fibril formation in protein solutions is reflected in increased β-sheet conformation and thus can be detected by means of circular dichroism (CD). We performed far-UV range CD measurements (190 to 260 nm) of HEWL solutions and investigated changes in their secondary structure content. Measurements were carried out on Jasco J-1500 CD spectrometer (Tokio, Japan) using a quartz cuvette with 0.1 cm path length. The buffer solutions were diluted to 10 mM concentration prior preparing HEWL solutions due to their absorption in the far-UV field. Fresh HEWL samples were diluted to 0.3 mg/mL, meanwhile agitated and static HEWL samples to 0.25 mg/mL. Obtained CD spectra were presented as mean residue ellipticity [θ], which is calculated with the subsequent equation:

(1) $$\left[\theta \right]=\frac{\theta \cdot 0.001\cdot M_{0}}{\gamma \cdot l}$$

where, θ, *l* and γ represent the measured ellipticity in milli-degrees, path length in cm and the HEWL concentration in mg/mL, respectively. $M_{0}$ denotes mean residue mass and for HEWL it was calculated to be 111,700 mg/mol [33]. HEWL secondary structure changes were estimated with the online server BeStSel [34].

### 2.7. Differential Scanning Calorimetry (DSC)

DSC is a thermoanalytical technique primarily used for investigating thermodynamics of conformational changes, stability and interactions of proteins and other biomolecules. Heat capacity changes of thermally induced protein denaturation reveal valuable information concerning changes of protein primary structure, their hydration and lastly non-covalent interactions of protein secondary and tertiary structure [35]. DSC thermograms were obtained using Nano DSC II (CSC). Protein solutions were diluted with appropriate buffer solutions to a final concentration of 3 mg/mL. Protein and buffer solutions were subsequently degassed for 15 min before injection in the DSC cells. DSC scans were recorded from 20 to 95 °C with a heating rate of 2 °C/min. Raw DSC data was analyzed with NanoAnalyze software, where we subtracted the corresponding buffer-buffer scans from protein data. Protein scans were normalized to total protein concentration to obtain thermograms of partial heat capacity as a function of temperature.

## 3. Results

### 3.1. HEWL Molecular Structure and Stability Depend on the Buffer Identity

**Figure 1:**
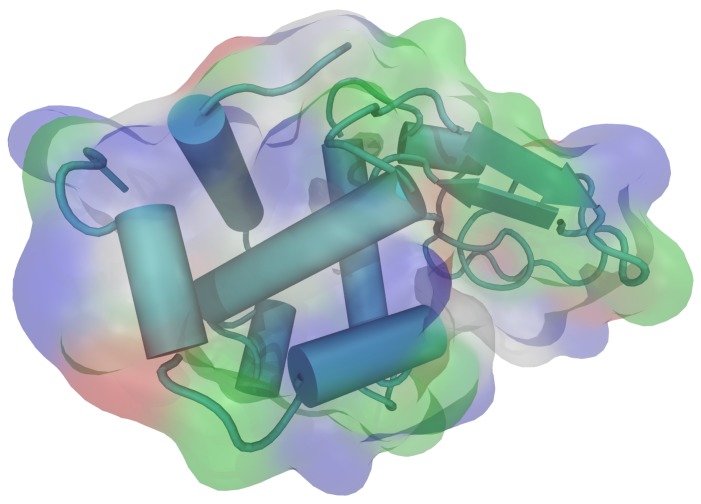
The three dimensional structure of hen egg-white lysozyme (HEWL) molecule with secondary structure elements, as visualized by VMD (PDB: 1aki.pdb) [38,39]. The colours on the molecular surface indicate the nature of the amino acid residues: acidic—red, basic—blue, polar—green, nonpolar—white.

HEWL consists of a single chain with 129 amino acid residues. It contains five alpha helical regions and five regions containing beta sheets, linked together by a number of beta turns and a large number of random coils [36]. All of the ionizable groups and most of the polar side chains (except for Gln57 and Ser91) are distributed over the surface of the molecule, while the majority of the nonpolar side chains lie within the protein interior. There are a number of hydrophobic groups on the surface, however, and they include most prominently Trp62, Trp63, Ile98, Val109, and Trp123 (Figure 1). The isoelectric point (pI) for the molecule is 11.35 [37], which means the protein molecule is carrying the net positive charge below this pH. This was the range of pH where the experiments were performed.

The net surface charge of a HEWL molecule at these different pH values (2.0, 3.0, 4.5, 7.0, 7.5, 8.0, 9.0 and 10.0) was calculated using the YASARA computational tool [40] with AMBER14 force field [41] and are given in Table 1.

The net surface charge is strongly positive (+18) at pH = 2.0 and 3.0, but reduces to +7 at pH = 7.0 and shows no more dependence on pH till the pH = 10.0 which is the highest pH at which the experiments were performed.

**Figure 2:**
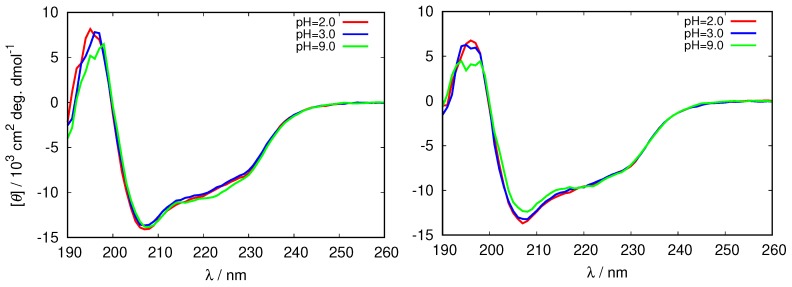
Circular dichroism (CD) spectra of fresh HEWL solutions in different 0.25 M glycine buffers (**left**), and 0.5 M glycine buffers (**right**).

We proceeded by examining the structure of the HEWL molecule in its reference state (i.e., where the fibrils were not yet formed) at different pH values by analyzing the far-UV circular dichroism (CD) spectra which are sensitive to conformational changes in proteins [42]. The results for the CD spectra of HEWL in glycine buffer that enabled us to study the protein at both, acidic and basic pH values are at two different ionic strength shown in Figure 2 (the complete set of spectra is given in Supplementary Material in Figure S1).

One can see that the bands at 208 nm, and 222 nm characteristic for α-helix, change with ionic strength, as well as with the pH of the solution. The negative ellipticity of these bands is largest in the 0.25 M buffer at pH = 2.0, and the smallest in the 0.5 M glycine at pH = 9.0, suggesting the decrease in the secondary structure of HEWL in this direction. This is in agreement with previous observations that the secondary structure of HEWL decreases with the increasing buffer concentration [43].

**Figure 3:**
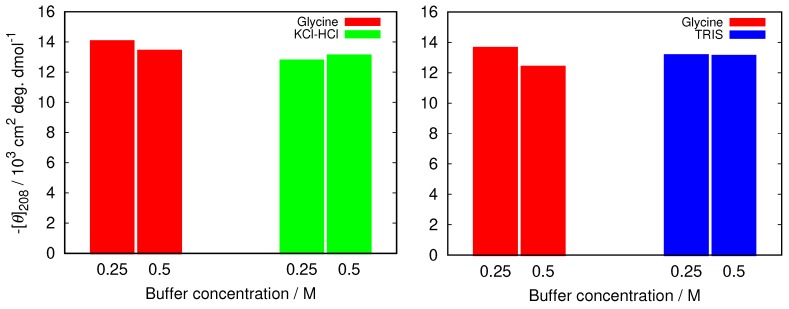
The comparison of negative ellipticity at 208 nm between glycine and two other buffers at different buffer concentrations at pH = 2.0 (**left**), and pH = 9.0 (**right**). The error in the values was estimated (as a standard deviation between different (at least two) sets of measurements) to be 1.5%.

To further examine how this depends on the buffer used we compared the decrease in the band at 208 nm in glycine buffer with the increased buffer concentration with other buffers at pH = 2.0 (KCl-HCl buffer), and at pH = 9.0 (TRIS buffer). The results in Figure 3 show that the changes are much less pronounced in other buffers, suggesting less protein stabilization in glycine buffer solutions.

A number of algorithms exist which use the data from far-UV CD spectra to provide an estimation of the secondary structure composition of proteins. We here used the BeStSel [34] software to estimate the amount of the β-antiparallel sheets in fresh HEWL molecules in different buffers, the structure that has a particular role in amyloid fibril formation. The β-results are given in Table 2.

Not much distinctive differences are noticed in secondary structure of the HEWL molecule; regardless of the buffer used the molecule contains approximately 27% of α-helix, and 12% of β-antiparallel sheet. A close inspection of the data would reveal that the least amount of β-antiparallel sheet is present in the 0.25 M glycine buffer at pH = 2.0, and increases with the pH, as well as with the buffer concentration. Interestingly, the secondary structure of the protein changes, not just with the ionic strength and pH, but with the buffer as well. There is much more β-antiparallel sheet structure present in the TRIS buffer, or KCl-HCl buffer of the same pH and ionic strength, compared to the glycine.

To inspect the protein surface characteristics, the fluorescence spectroscopy was applied. We used an extrinsic dye, 1-anilinonaphthalene-8-sulfonate (ANS) which interacts noncovalently with proteins, and namely with its exposed hydrophobic and charged groups [32]. While ANS is hardly fluorescent in aqueous environment its interaction with hydrophobic binding sites is accompanied by an increase in fluorescence, as well as with a blue shift of the peak maximum [32]. Based on that a method has been established to quantitatively assess the relative surface hydrophobicity from the association constant of ANS-protein for certain proteins [44].

**Figure 4:**
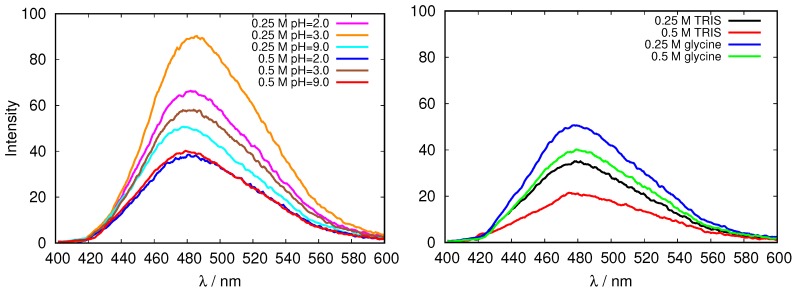
Emission fluorescence of 1-anilinonaphthalene-8-sulfonate (ANS) in fresh HEWL solutions in glycine buffers of different concentrations, and at different pH values (**left**), and the comparison of emission fluorescence of ANS in fresh HEWL solutions at pH = 9.0 for two different buffers, tris(hydroxymethyl)aminomethane (TRIS) and glycine (**right**).

The results for the fluorescence emission of 200 μM ANS excited at 380 nm in glycine and TRIS buffer solutions of fresh HEWL are presented in Figure 4. One can see that the intensity of the fluorescence decreases with the increasing ionic strength, however, no simple relationship between the buffer pH and the fluorescent intensity, can be detected. A possible reason for this could be the fact that both, hydrophobic, as well as ionic environment can contribute to the fluorescence intensity [32] in our case, which makes it impossible to estimate the change of the surface hydrophobicity upon change in pH using this method in this case. Further, the characteristics of the protein surface as reflected in emission fluorescence of ANS clearly depend on the buffer (Figure 4, right).

**Figure 5:**
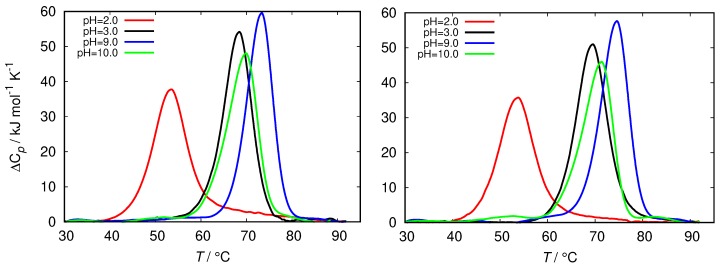
Thermograms of fresh HEWL at different ionic strength of glycine buffer, 0.25 M (**left**) and 0.5 M (**right**).

The differential scanning calorimetry (DSC) method is commonly used to characterize the thermal stability of the proteins. However, since the latter strongly depends on the protein interactions with the surrounding solvent it can be indirectly used to estimate the surface hydrophobicity of the protein [45]. The latter is related to the change in the heat capacity upon denaturation of the protein. Figure 5 shows the comparison of the DSC thermograms of HEWL solutions in glycine buffers of different concentrations at different pH. The fresh HEWL in 0.5 M and 0.25 M glycine buffer at pH = 2.0 displays a greatly decreased conformational stability in comparison with glycine buffers at other pH, as well with other buffers (Figure S2). The melting temperature ($T_{m}$) of these samples is almost 20 °C lower than for HEWL in other buffer solutions, which are mutually within 6 °C.

To summarize, in view of the current picture that the buffer influences the protein structure and stability by binding to its surface [16,17,18], the glycine buffer at low pH, where the HEWL molecule is highly positively charged has, itself in the cationic form, the lowest tendency to bind to the protein surface. This is reflected in destabilization of the protein structure upon increasing the ionic strength, as well as upon increasing temperature. No such pronounced effects have been observed for other pH values or other buffers.

### 3.2. The Amyloid Fibril Formation

While it has been originally established that HEWL can form amyloid aggregates by heating the protein solution at acidic pH, acidic hydrolysis providing amyloidogenic core peptides, it’s been shown recently that HEWL can form amyloid fibrils even at pH = 2.0 and 37 °C, under agitated, as well as static incubating conditions [6]. Since we were interested in the mechanism of amyloid fibril formation in nature, where no hydrolysis occurs, we conducted the study at these conditions. After the expired incubation periods HEWL solutions were examined for the presence of amyloid aggregates by analysing the amyloid-like thioflavin T (ThT) and Congo red binding affinities.

**Figure 6:**
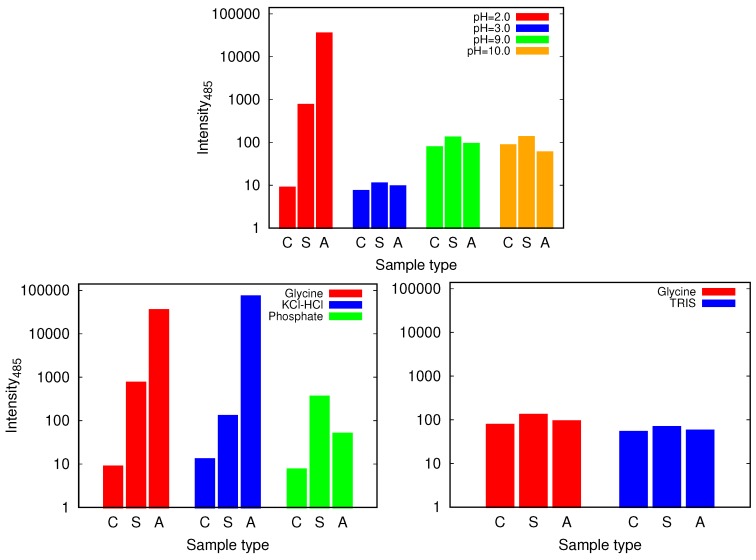
The intensity of thioflavin T (ThT) fluorescence emission at 485 nm for control (C), static (S) and agitated (A) samples of HEWL in 0.5 M glycine buffer solutions (**top**) and its comparison with 0.5 M KCl-HCl (pH = 2.0), phosphate (pH = 2.0) and TRIS (pH = 9.0) buffer solutions **(bottom**). Note that the scale is logarithmic. For the higher pH values of glycine higher intensities are observed for all the samples which is due to different binding affinity of ThT at different pH values.

We proceed with the discussion of the glycine buffer that enabled us the comparison of the results at the acidic and basic pH range. Although all the samples show some kind of increase of ThT binding affinities as reflected in the fluorescence emission intensity that occurs when the dye binds to the cross-β structure of the amyloid fibrils [30], the only markedly pronounced change, suggesting amyloid fibril formation occurs for agitated samples at pH = 2.0 (Figure 6 and Figure S3). However, the described conditions are not enough for the amyloid fibrils to form; no indication of amyloid fibrils have been found in glycine buffer of low enough concentration (0.25 M), or, for example, in phosphate buffer.

**Figure 7:**
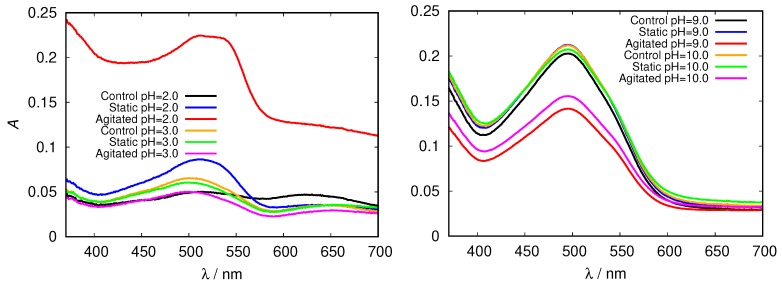
Congo red absorption spectra of HEWL in acidic (**left**) and alkaline (**right**) range of 0.5 M glycine buffer solutions.

These findings were further confirmed by measuring the absorption spectra of HEWL solutions upon addition of Congo red dye. Its binding mechanism is still not well understood, but it mostly depends on the protein secondary structure, predominantly binding to cross-β sheet structure. Binding of Congo red to amyloid fibrils induces a red shift in its maximum absorbance wavelength from 490 to 540 nm and/or leads to an increase in sample absorbance [29]. Again, we first inspected the spectra for glycine buffer solutions of different pH and different concentration. Clear differences in peak position (red shift), intensity and shape in comparison with the control fresh HEWL solution is observed in acidic pH; a major increase in Congo red absorbance is observed in the agitated HEWL at pH = 2.0 in 0.5 M glycine buffer solution (Figure 7).

In the basic range of glycine no fibrils were detected; relatively high absorption intensities are due to different Congo red absorption properties at these conditions, as the dye adopts a red coloured sulphonazo form above pH = 5.0 [46].

**Figure 8:**
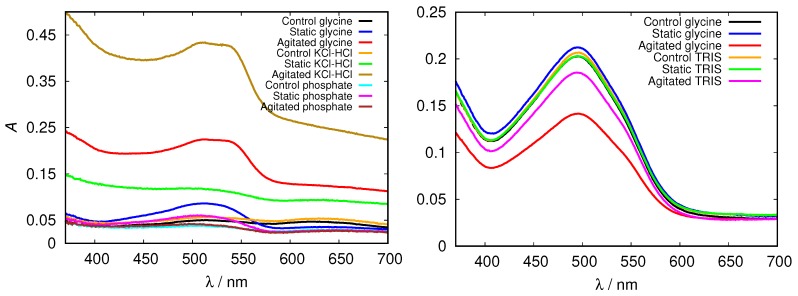
Congo red absorption spectra of HEWL at pH = 2.0 (**left**), and pH = 9.0 (**right**) in 0.5 M glycine buffer solutions, compared to KCl-HCl, phosphate, and TRIS buffer solutions, respectively.

Again we tested the buffer specificity by comparing the results for HEWL in glycine buffer solutions with those in other buffers at same pH and buffer concentration, namely with KCl-HCl and phosphate buffer at pH = 2.0 and with TRIS buffer at pH = 9.0 (Figure 8 and Figure S4). The results show that not much difference in the Congo red absorption is detected among different buffer solutions in the basic range of pH values. Similar as already suggested by the ThT fluorescence emission, however, at pH = 2.0 the differences between buffers become more evident: the amyloid fibrils form in the glycine and KCl-HCl buffer at high enough concentration, but not in the phosphate buffer at the same pH. Note that the required buffer concentration for amyloid fibrils to form is much lower in KCl-HCl buffer compared to the glycine buffer, suggesting a certain level of protein stabilization by the latter.

**Figure 9:**
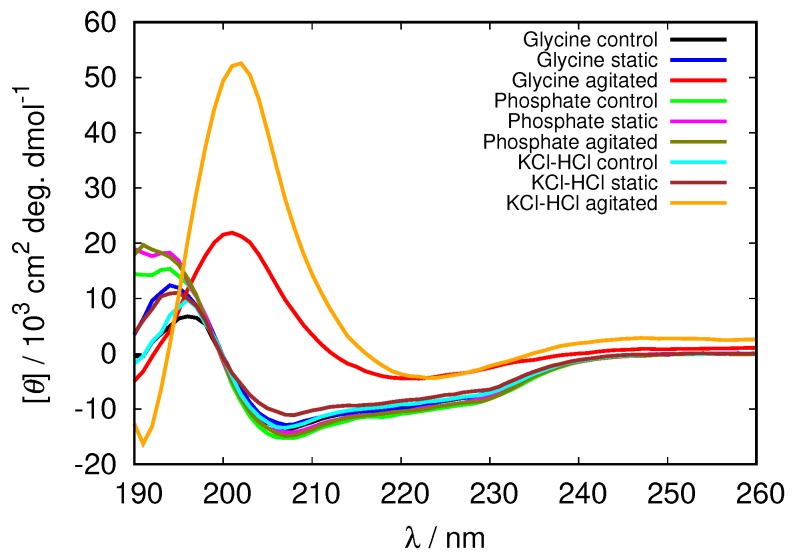
CD spectra of HEWL at pH = 2.0 in 0.5 M glycine buffer solutions compared to 0.5 M phosphate and KCl-HCl buffer solutions.

To analyze the level of protein denaturation upon fibrillization the CD spectra of all the solutions were recorded and analyzed using BeStSel software. Although some minor changes can be observed in the spectra in all cases (Figures S5 and S6), the only distinctive changes, suggesting the fibrillization occur at pH = 2.0 in agitated solutions (Figure 9). In glycine and KCl-HCl buffer solutions of high enough concentration at this pH the negative band at 208 nm, characteristic for α-helix completely disappears, while a strong positive band at approximately 200 nm appears, indicating the increased amount of β-structure in the HEWL molecule; the molecule under these conditions contains 87% of β-antiparallel sheet in 0.5 M glycine buffer, and over 90% of β-antiparallel sheet in KCl-HCl concentrated buffers (Table 3). No noticeable changes in the secondary structure were observed in the phosphate buffer (secondary structure content results for other buffer solutions are included in Supplementary Material Tables S1–S3).

### 3.3. Electrostatic Screening Facilitates Fibrillization

The results we obtained studying the fibrillization process of HEWL in different buffers indicate that the concentration of the buffer also plays a role in stabilization of the protein solutions; the amyloid fibrils were, for example, detected in the 0.5 M glycine buffer at pH = 2.0, but not in the 0.25 M buffer solutions of the same buffer, while in the KCl-HCl buffer of the same pH the amyloids were formed even in 0.25 M buffer solutions. To test the idea of importance of electrostatic screening we attempted to initiate the fibrillization process by increasing the electrostatic screening while maintaining the same ionic strength of the buffer solution. As no amyloid fibrils were formed in 0.25 M glycine buffer at pH = 2.0, we gradually replaced some of the buffer solution with NaCl solution (the concentration of NaCl in the buffer solution was 10, 30, 50, and 70 mM). The solutions were, again inspected by fluorescence and absorption spectroscopy, as well as CD spectra analysis.

**Figure 10:**
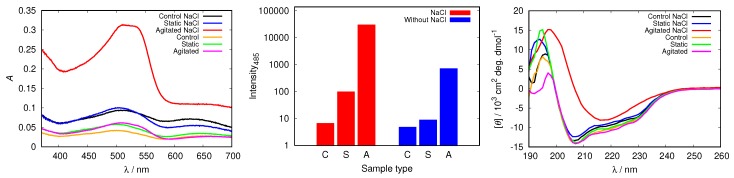
Congo red absorption spectra of HEWL (**left**), the intensity of ThT fluorescence emission at 485 nm for control (C), static (S) and agitated (A) samples of HEWL (**middle**), and CD spectra of HEWL (**right**), compared in 0.25 M glycine solutions with and without 50 mM NaCl. Note the scale for ThT intensity is logarithmic.

In all cases studied, the addition of NaCl triggered fibrillization in agitated samples at pH = 2.0 (Figure 10 and Figure S7). The spectra of Congo red in the agitated sample at pH = 2.0 (with 50 mM NaCl) presented in Figure 10 display a substantially increased absorption intensity, which indicates fibril formation. The process is further confirmed by the increased fluorescence intensity of ThT, as well as with CD spectra.

To examine other possible effects of NaCl, except electrostatic screening, on the protein conformation, we repeated the analysis for glycine buffers at different pH values, as well as with other buffer solutions, but no effect of NaCl was detected (Figure S8).

### 3.4. Binding Inert Molecules Inhibits HEWL Fibrillization

Other inert molecules, besides for buffer components can also interact with protein molecules in the solution. One of the most common excipients in protein formulations is polyethylene glycol (PEG). Recently it has been reported that in contrast with common beliefs, PEG can interact with a protein molecule, and bind to its surface, in some cases even causing conformational changes [47].

**Figure 11:**
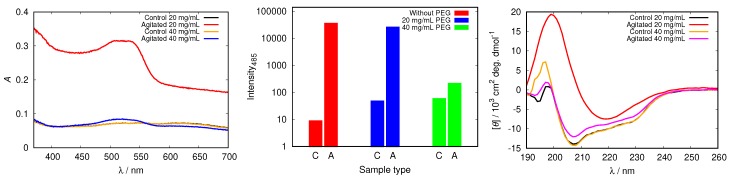
Congo red absorption spectra of HEWL (**left**), the intensity of ThT fluorescence emission at 485 nm for control (C), static (S) and agitated (A) samples of HEWL (**middle**), and CD spectra of HEWL (**right**) in 0.5 M glycine at pH = 2.0 with different concentrations of added PEG12000. Note the scale for ThT intensity is logarithmic.

To test the inhibitory capabilities of other, not buffer, molecules to stabilize protein molecules by binding to its surface we added different concentrations of PEG12000 to our HEWL solutions. Figure 11 demonstrates that adding 20 mg/mL of PEG12000 can not prevent HEWL from fibrillizing under agitated conditions, as the absorption peak for Congo red in this sample exhibits a red shift and increased absorbance in comparison with control and static samples. Meanwhile, the absorption spectra of samples at 40 mg/mL PEG12000 and above show no essential differences among samples with diverse incubation, thus denoting no fibrils were formed. With increasing PEG concentration the onset of HEWL fibrillization was arrested (Figure S9). Fluorescence results in Figure 11 support the trend obtained by Congo red. The intensity for the agitated sample with the lowest PEG12000 concentration (e.g., 20 mg/mL) was still high and only slightly lower than in the agitated sample without any PEG. However, the emission fluorescence decreased with increasing PEG concentration (Figure 11). The CD spectra presented in Figure 11 further confirm that amyloid fibrils are present only in the agitated sample with lowest (20 mg/mL) PEG concentration, as solely this sample displays a distinct single minimum for the cross-β sheet secondary structure. With rising PEG concentration static and agitated samples show only small decreases in α-helix content. Adding PEG to aqueous solutions also influences its viscosity; the viscosity of solutions with PEG12000 concentration 40 mg/mL is approximately three times higher than that of buffer solutions without PEG [48]. This could in principle influence the kinetics of fibril formation. Never-the-less, the CD spectra of samples with added PEG clearly show that no changes in protein structure occur if the concentration of PEG is high enough, confirming the protective role of these molecules in protein solutions.

## 4. Discussion

Interactions in biological systems, which include aqueous protein solutions, are very complex, therefor their interpretation is often challenging. All experimental techniques can be subjected to false positive, as well as false negative results, so the presence of fibrils in our solutions was confirmed on the basis of three complementary techniques. From all the analyzed HEWL buffer solutions it is clear that amyloid fibrils were formed only under agitated conditions at pH = 2.0 in certain buffers and at sufficient ionic strength. This confirms previous findings that buffer pH value plays an important role in assuring appropriate solution conditions for the growth of amyloid fibrils. A clear indication of the pH importance is visible in 0.5 M glycine solutions, where no fibrils were formed in the basic pH range and also at acidic pH = 3.0. Whereas at pH = 2.0, we could observe gel-like turbid solutions with a certain presence of amyloid fibrils, no such changes in the solution were observed at other pH values. Such harsh solution conditions were necessary to provoke partial unfolding of HEWL molecules, which has already been proposed to have an important role in protein fibrillization [49].

To achieve the fibrillization, the solutions needed to be agitated under incubating conditions for a substantial period of time, suggesting the importance of the kinetic, not just thermodynamic conditions of the process. It is important to note that at pH = 2.0 HEWL molecule has an estimated surface net charge of +18, which is much higher than at other pH values (Table 1). This produces significant electrostatic repulsion between protein molecules and thus creates an energy barrier towards HEWL molecules association. Agitation is one possibility to overcome these kinetically unfavourable conditions [6] (another options include additions of salt to screen the positive charges and incubation at high temperatures above 50 °C [50,51]). It provides more readily formed specific contacts between protein molecules, necessary to form the aggregates. Petkova et al. reported of another feature of agitated solutions: the accelerated formation of fibrils due to disassembly of mature long amyloid aggregates. Formed small fragments with additional free ends could enable quicker recruitment of monomers that form fibrils [52,53], in this way accelerating the fibrillization process.

Nonetheless, our results show that merely pH does not dictate the fibrillization process; even though the amyloid fibrils were formed in 0.5 M glycine buffer at pH = 2.0, no amyloid fibrils were detected in phosphate buffer of the same ionic strength and at the same pH value. This observation points toward buffer specific effects, which are common in biological systems [21]. Buffers were found to influence all three aspects of physical stability of proteins: conformational, colloidal and interfacial [17]. Conformational stability is usually linked with the temperature of denaturation ($T_{m}$) which can be significantly altered by binding of buffer ions to the protein molecules. In our study we could observe a greatly diminished conformational stability of HEWL in glycine solutions at pH = 2.0 and amplified stabilization with, for example phosphate buffer in which no fibrillization process occured (Figure 5). One should take into account, however, that simple ions that do not have specific binding to the protein surface can also increase protein thermal stability through their effect on water structure, increasing its surface tension [54], but at the same time cannot influence other aspects of protein stability. The colloidal stability which is governed by electrostatic interactions, is also dictated by buffers, since their pH value determines the overall charge of the protein molecule. However, it has been observed that some buffers can surpass the effect of pH and accumulate on protein surface, consequently lowering their colloidal stability [17,55]. Interfacial damage caused during sample agitation could also play an important role in fibril formation of HEWL. Small molecules, in this case buffer ions, can bind to the protein surface, and act as a protective coating, stabilizing the native structure of the protein [16,43,56].

Other small molecules that interact with the protein surface can stabilize the protein native structure as well. PEG molecules that have been since recently mostly used as an inert crowding agent and were known as an additive for protein refolding [57], have been shown to have the ability to bind to the protein surface and affect its conformational stability [47]. Although it has been observed that PEG molecules of high enough molecular weight can trigger partial unfolding of the protein, no such effects on the HEWL native structure have been observed in our study. However, adding PEG12000 of 40 mg/mL or higher concentration to the glycine buffer solution suppresses the amyloid fibril formation.

The importance of the electrostatic interactions in the solutions is usually regulated by changing the concentration of a simple electrolyte present. At high enough concentrations of the latter the electrostatic interaction can be efficiently screened reducing the colloidal stability of the solutions [25]. This colloidal destabilization plays an important role in the fibrillization process as well. Despite the fact that we did not observe the formation of fibrils in 0.25 M glycine buffer solution at pH = 2.0, the fibrillization has taken place upon addition of different concentrations of NaCl. By doing that we reduced the amount of buffer in the solution while at the same time increased the electrostatic screening by the presence of simple ions. The net salt effect is usually determined by the competition between ion adsorption on the protein surface and exclusion forces due to the ion preference for hydration [21]. In our HEWL solutions slightly chaotropic chloride anions can preferentially bind to chaotropic lysine and arginine residues, reducing the surface charge of the protein. The effective charge density, which is determined by the number of condensed counterions on protein surface, can considerably affect interparticle interactions between proteins and thus favour or prevent essential contacts for fibril formation. At the same time, by replacing some of the buffer molecules at the protein surface the simple salt ions additionally destabilize the protein molecule. The addition of salt could not trigger HEWL fibrillization at other pH values, suggesting the importance of the partial unfolding of the HEWL molecule at pH = 2.0 in the initial stage of the process.

It is without doubt that protein aggregation is a multistage complicated process, with amyloid fibril formation being no exception. Amyloid monomers first unite into small oligomers of similar size. After oligomers reach a certain critical concentration the nucleation of protofibrils commences. They grow as polymers of oligomers and after elongating above a few hundred nanometers, those protofibrils seem to self-assemble into even longer and stiffer mature amyloid fibrils [58]. For this reason their inhibition is at most complex and yet unsolved, since it can occur at different stages of this process. So far small molecule compounds, such as 4-amino-phenol and 2-amino-4-chlorophenol, were already reported to disrupt human lysozyme and HEWL fibrils, due to their destabilizing effect on hydrogen bonds forming β-sheets in the core of fibrils and hydrophobic interactions between amino acid side chains as well [59]. Here we show that small molecules, such as buffer components, or PEG that bind to the protein surface can stabilize the native structure of the protein, suppressing the fibrillization process in its initial stage. Crowding environment can also play a role in stabilizing protein solutions. Although several studies report of accelerated amyloid growth after induced crowding in protein solutions [60,61], some cosolutes, such as sugars and proteins, were proposed to inhibit protein fibrillization [2,62]. Crowding agents can stabilize the compact native structure of the protein and obstruct its further association into oligomers.

## 5. Conclusions

In this study, we investigated how different solution conditions influence the fibrillization of hen egg-white lysozyme (HEWL). Amyloid fibrils of HEWL formed only under agitated conditions at pH = 2.0 where the conditions in the solution facilitated the partial unfolding of the protein, as well as favourable kinetic conditions to overcome the electrostatic repulsion barrier between highly positively charged molecules. The amyloid fibrils under these conditions only formed in 0.5 M glycine buffer, and in 0.25 and 0.5 M KCl-HCl buffers. The phosphate buffer at these conditions did not facilitate the fibrillization process, suggesting the importance of buffer specific effects. Several other buffer solutions, such as TRIS, acetate, HEPES and cacodylate were also recognized as stabilizing buffers, which all hindered the onset of HEWL fibrillization. To commence amyloid fibril formation in at first non-amyloid solution conditions a simple electrolyte, NaCl, was combined with glycine to reach an ionic strength of 0.25 M. This induced the fibrillization in agitated solutions of 0.25 M glycine buffer at pH = 2.0, but not in other solutions. In an attempt to prevent HEWL from aggregating into fibrils an inert polymer, PEG12000, was added to 0.5 M glycine at pH = 2.0, where otherwise amyloid fibrils were formed. Above a certain concentration PEG prevented HEWL from associating into oligomers, and suppressed fibrillization. We concluded that PEG molecules, similar as some stabilizing buffer components interact with the surface of protein molecules, stabilizing their native conformation. Since buffers are essential components in protein formulations the possible stabilization of protein molecules by buffer molecules can avoid using other additives to the systems. The possible mechanism of their acting can further serve as a guideline to developing new, better biological buffers, as well as other ligands to prevent fibrillization. Even though this study can provide a useful insight in that direction, the exact mechanism of amyloid fibril formation still remains to be uncovered.

## Figures and Tables

**Table 1:** Net charge on hen egg-white lysozyme (HEWL) surface calculated with the YASARA computational tool by using AMBER14 force field [41].

pH Value	Net Charge	Positive Amino Acids	Negative Amino Acids
2.0	+18.0	11 Arg, 6 Lys, 1 His	/
3.0	+18.0	11 Arg, 6 Lys, 1 His	/
4.5	+8.0	11 Arg, 6 Lys, 1 His	7 Asp, 2 Glu, 1 Leu
7.0	+7.0	11 Arg, 6 Lys	7 Asp, 2 Glu, 1 Leu
7.5	+7.0	11 Arg, 6 Lys	7 Asp, 2 Glu, 1 Leu
8.0	+7.0	11 Arg, 6 Lys	7 Asp, 2 Glu, 1 Leu
9.0	+7.0	11 Arg, 6 Lys	7 Asp, 2 Glu, 1 Leu
10.0	+7.0	11 Arg, 6 Lys	7 Asp, 2 Glu, 1 Leu

**Table 2:** Estimated secondary structure content (%) of fresh HEWL in different buffer solutions. The error estimated from two different sets of measurements was estimated to be ±2%.

Solution	α-Helix	β-Antiparallel Sheet	β-Parallel Sheet	β Turn
0.5 M glycine, pH = 2.0	24	12	3	16
0.25 M glycine, pH = 2.0	29	8	3	11
0.5 M glycine, pH = 3.0	23	15	4	13
0.25 M glycine, pH = 3.0	28	9	2	10
0.5 M glycine, pH = 9.0	28	11	4	15
0.25 M glycine, pH = 9.0	30	10	1	11
0.5 M glycine, pH = 10.0	28	14	2	18
0.25 M glycine, pH = 10.0	31	12	1	13
0.5 M TRIS, pH = 7.0	24	12	5	14
0.25 M TRIS, pH = 7.0	28	10	1	11
0.5 M TRIS, pH = 7.5	25	9	4	16
0.25 M TRIS, pH = 7.5	29	10	1	13
0.5 M TRIS, pH = 8.0	25	10	5	15
0.25 M TRIS, pH = 8.0	29	14	3	14
0.5 M TRIS, pH = 9.0	31	13	2	17
0.25 M TRIS, pH = 9.0	30	14	0	14
0.25 M cacodylate, pH = 7.0	24	13	5	16
0.5 M cacodylate, pH = 7.0	27	15	2	16
0.1 M KCl-HCl, pH = 2.0	24	11	3	16
0.5 M KCl-HCl, pH = 2.0	23	12	2	15
0.25 M KCl-HCl, pH = 2.0	24	15	3	15
0.5 M Phosphate, pH = 2.0	30	7	2	15
0.5 M Phosphate, pH = 7.0	25	11	4	16

**Table 3:** Estimated secondary structure content (%) of control (C), static (S) and agitated (A) HEWL in different buffer solutions at pH = 2.0 at different ionic strength. The error estimated from two different sets of measurements was estimated to be ±2%

Solution	α-Helix	β-Antiparallel Sheet	β-Parallel Sheet	β Turn
0.5 M glycine (C)	24	12	3	16
0.5 M glycine (S)	23	10	5	15
0.5 M glycine (A)	2	87	0	11
0.25 M glycine (C)	29	8	3	11
0.25 M glycine (S)	28	4	6	12
0.25 M glycine (A)	33	15	0	12
0.1 M KCl-HCl (C)	24	11	3	16
0.1 M KCl-HCl (S)	22	13	3	15
0.1 M KCl-HCl (A)	28	18	10	11
0.25 M KCl-HCl (C)	24	15	3	15
0.25 M KCl-HCl (S)	21	12	5	16
0.25 M KCl-HCl (A)	0	99	0	2
0.5 M KCl-HCl (C)	23	12	2	15
0.5 M KCl-HCl (S)	19	18	5	14
0.5 M KCl-HCl (A)	0	93	0	7
0.5 M phosphate (C)	30	7	2	15
0.5 M phosphate (S)	28	6	3	16
0.5 M phosphate (A)	30	6	2	15

## Supplementary Materials

The following are available at http://www.mdpi.com/2218-273X/9/2/65/s1.

## Abbreviations

The following abbreviations are used in this manuscript:

HEWL	Hen egg-white lysozyme
CD	Circular dichroism
DSC	Differential scanning calorimetry
ThT	Thioflavin T
TRIS	tris(hydroxymethyl)aminomethane
HEPES	2-[4-(2-hydroxyethyl)piperazin-1-yl]ethanesulfonic acid
ANS	8-(Phenylamino)-1-naphthalenesulfonic acid
PEG	Polyethylene glycol
