# Supplementary material for: The Role of Buffers in Wild-Type HEWL Amyloid Fibril Formation Mechanism
Source: Biomolecules. 2019 Feb 14;9(2):65. doi: 10.3390/biom9020065 (PMC6406783; doi:10.3390/biom9020065)
Supplement: Supplementary file 1 [file biomolecules-09-00065-s001.pdf]

Supplementary Materials: The role of buffers in wild-type HEWL amyloid fibril formation mechanism

Sandi Brudar [1], Barbara Hribar-Lee [1,*]

The results of our research from multiple techniques were processed as follows:

From raw data (absorption spectra) obtained with UV-Vis measurements we have depleted the corresponding Congo red reference solutions (only dye + buffer). This process was performed with Microsoft Excel and the obtained data were then plotted with Gnuplot 5.0. The raw flourescence emission spectra were manipulated in a similar way. Cor- responding dye-buffer baselines (only dye + buffer) were subtracted from raw data and the intensity was corrected due to different sample dilution (agitated samples were considerably diluted in order to obtain results within the working range of the instrument). Afterwards the data vas visualized with Gnuplot 5.0 Raw ellipticity data from circular dichroism measurements were converted to molar ellipticity, meaning the results were normalized to protein concentration, path length and mean residue mass. The obtained CD spectra were plotted with Gnuplot 5.0. Secondary structure content was evaluated with the BeStSel server, which is freely accessible online. Detailed information of how the protein sec- ondary structure is calculated was published in Micsonai, A.; Wien, F.; Kernya, L.; Lee, Y.; Goto, Y.; Refregiers, M.; Kardos, J. Accuarate secondary struc- ture prediction and fold recognition for circular dichroism spectroscopy. PNAS 2015,112, E3095–E3103.doi:10.1073/pnas.1500851112. DSC data was analyzed with NanoAnalyze software. We subtracted the corresponding buffer-buffer scans from raw protein data and normalized them to protein concentration and molecular weight. The processed thermograms were then also displayed with Gnuplot 5.0.

All the measurements were repeated at least twice and the error was evaluated as a standard deviation between (at least) two different sets of measurements. The differences between the sets of measurements (standard deviation) were smaller than the thickness of the lines in graphs and were thus not shown.

Our conclusions (assumptions) on the role of buffer specific effects in the fibrillization process of HEWL are based on the results obtained from several complementary techniques, as discussed in section 3. The results of different techniques are consistent among each other, as they are consistent with previously published results (see references) on fibrillization of this protein.

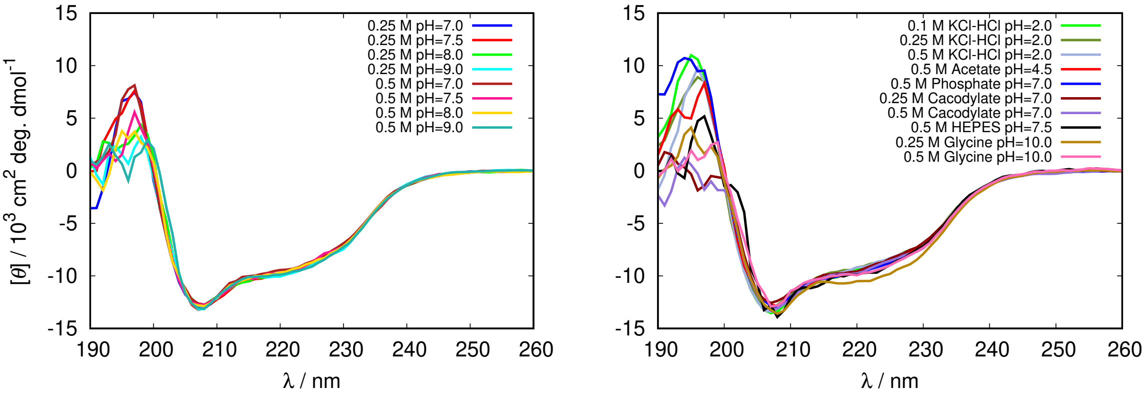

Figure S1. CD spectra of fresh HEWL solutions in TRIS buffers (left) and other buffers (right). There are no significant alterations between the CD spectra of fresh HEWL in different buffer solutions.

Table S1. Estimated secondary structure content (%) of control, static and agitated HEWL in glycine buffer solutions at different ionic strength and pH values. The error estimated from two different sets of measurements was estimated to be ±2%.

Solution	α-helix	β-antiparallel sheet	β-parallel sheet	β turn
0.5 M control, pH=3.0	23	15	3	13
0.5 M static, pH=3.0	26	12	4	14
0.5 M agitated, pH=3.0	24	13	4	16
0.5 M control, pH=9.0	28	11	4	15
0.5 M static, pH=9.0	25	11	4	15
0.5 M agitated, pH=9.0	27	11	4	14
0.5 M control, pH=10.0	28	14	2	17
0.5 M static, pH=10.0	23	11	6	14
0.5 M agitated, pH=10.0	21	15	4	16
0.25 M control, pH=3.0	28	9	2	10
0.25 M static, pH=3.0	26	4	8	14
0.25 M agitated, pH=3.0	26	4	7	13
0.25 M control, pH=9.0	30	10	1	11
0.25 M static, pH=9.0	28	3	8	14
0.25 M agitated, pH=9.0	27	5	6	13
0.25 M control, pH=10.0	31	12	1	13
0.25 M static, pH=10.0	32	12	0	13
0.25 M agitated, pH=10.0	33	10	0	11

Table S2. Estimated secondary structure content (%) of control, static and agitated HEWL in TRIS buffer solutions at different ionic strength and pH values. The error estimated from two different sets of measurements was estimated to be ±2%.

Solution	α-helix	β-antiparallel sheet	β-parallel sheet	β turn
0.5 M control, pH=7.0	24	12	5	14
0.5 M static, pH=7.0	21	13	5	15
0.5 M agitated, pH=7.0	23	15	5	14
0.5 M control, pH=7.5	25	9	4	16
0.5 M static, pH=7.5	22	13	5	14
0.5 M agitated, pH=7.5	25	14	3	15
0.5 M control, pH=8.0	25	10	4	15
0.5 M static, pH=8.0	24	10	5	15
0.5 M agitated, pH=8.0	21	13	6	15
0.5 M control, pH=9.0	31	13	2	17
0.5 M static, pH=9.0	23	13	4	15
0.5 M agitated, pH=9.0	26	13	4	14
0.25 M control, pH=7.5	29	10	1	12
0.25 M static, pH=7.5	26	7	6	13
0.25 M agitated, pH=7.5	25	6	7	13
0.25 M control, pH=8.0	29	13	3	14
0.25 M static, pH=8.0	29	8	2	13
0.25 M agitated, pH=8.0	30	18	0	13
0.25 M control, pH=9.0	30	14	0	14
0.25 M static, pH=9.0	30	11	2	14
0.25 M agitated, pH=9.0	28	12	1	14

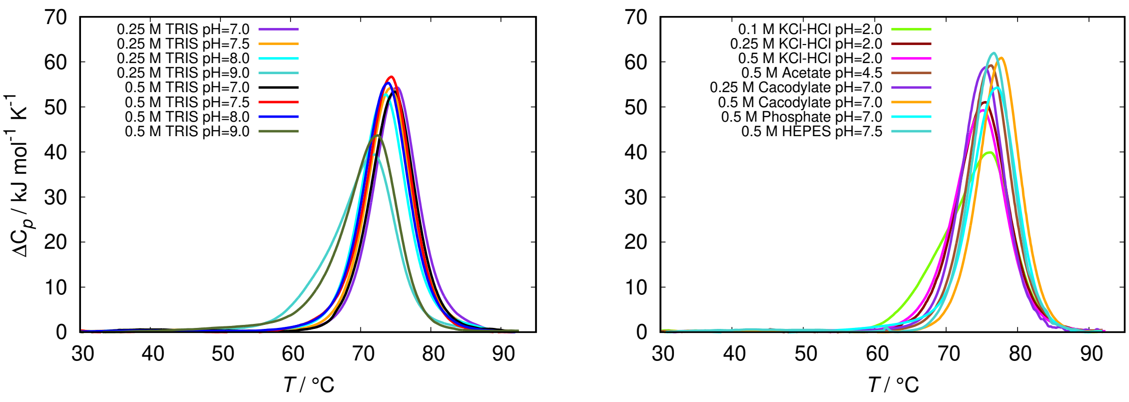

Figure S2. Thermograms of fresh HEWL in TRIS buffers (left) and other buffers (right). The conformational stability of fresh HEWL is very similar in all buffer solutions displayed here. The melting temperatures of HEWL solutions are mutually within 6 °C.

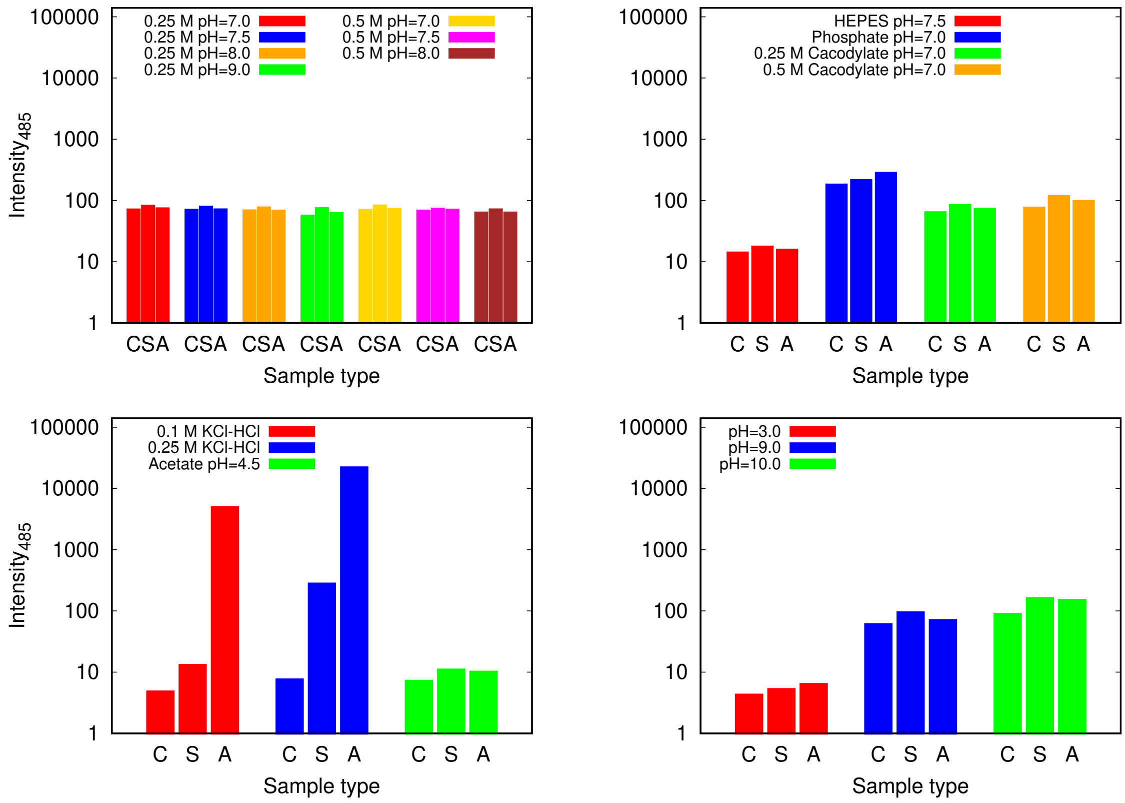

Figure S3. The intensity of ThT fluorescence emission at 485 nm for control (C), static (S) and agitated (A) samples of HEWL in different TRIS buffers (top left), 0.5 M HEPES, phosphate and different cacodylate buffers (top right), 0.5 M acetate and different KCl-HCl buffers (bottom left) and 0.25 M glycine buffers at different pH (bottom right). Note the scale is logarithmic. We can observe no major differences in the intensity of ThT in TRIS buffer solutions at different pH. Similar is the case with HEPES, cacodylate and phosphate in the basic range, where no essential differences among samples with diverse incubation can be seen. For the agitated sample in 0.1 M KCl-HCl there is a substantial increase in emission intensity of ThT, which indicates significant changes in HEWL structure, although no major amount of fibrils was confirmed by the low extent of β-antiparallel sheet in this sample (Table 3 of the manuscript). Meanwhile, the presence of fibrils was confirmed in the 0.25 M KCl-HCl, which displays an even higher emission intensity of ThT. For 0.5 M acetate and 0.25 M glycine buffer solutions at different pH no significant differences among samples with diverse incubation were observed, denoting no fibrils are present.

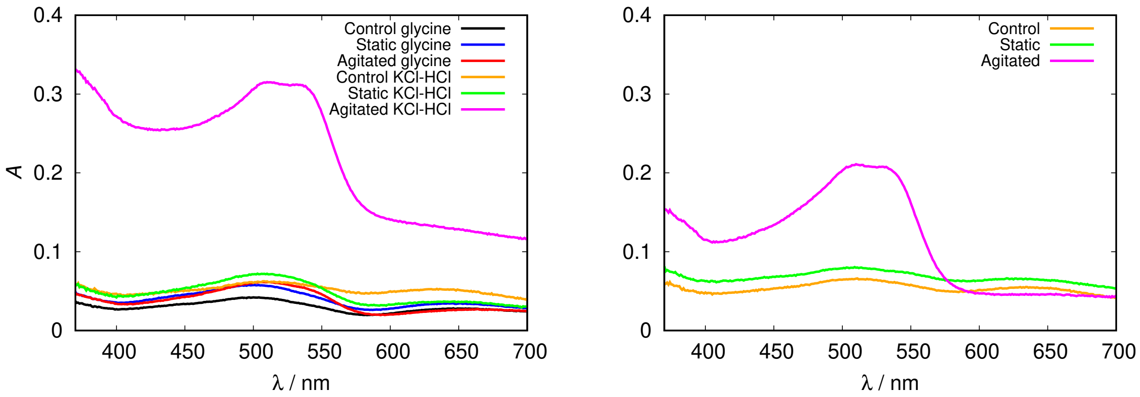

Figure S4. Congo red absorption spectra of HEWL in 0.25 M glycine and KCl-HCl buffer solutions at pH=2.0 (left) and in 0.1 M KCl-HCl at pH=2.0 (right). A major increase in Congo red absorbance is observed in the agitated HEWL at pH=2.0 in 0.25 M KCl-HCl buffer solution, denoting to fibrillization in this sample. For Congo red in 0.25 M glycine solutions no essential changes are observed among samples with diverse incubation. Although the peak for the agitated sample in 0.1 M KCl-HCl buffer solution clearly differentiates from other samples in intensity, shape and position no major quantity of fibrils was detected.

Table S3. Estimated secondary structure content (%) of control, static and agitated HEWL in different buffer solutions. The error estimated from two different sets of measurements was estimated to be ±2%.

Solution	α-helix	β-antiparallel sheet	β-parallel sheet	β turn
0.5 M Acetate pH=4.5				
control	23	12	4	14
static	25	11	3	14
agitated	25	15	3	14
0.5 M Phosphate pH=7.0				
control	25	11	4	16
static	25	12	5	13
agitated	25	14	5	14
0.5 M HEPES pH=7.5				
control	35	24	0	18
static	28	10	4	16
agitated	27	11	5	13
0.25 M Cacodylate pH=7.0				
control	24	13	5	16
static	24	13	4	13
agitated	25	16	3	16
0.5 M Cacodylate pH=7.0				
control	27	15	2	16
static	29	6	3	16
agitated	28	11	3	16

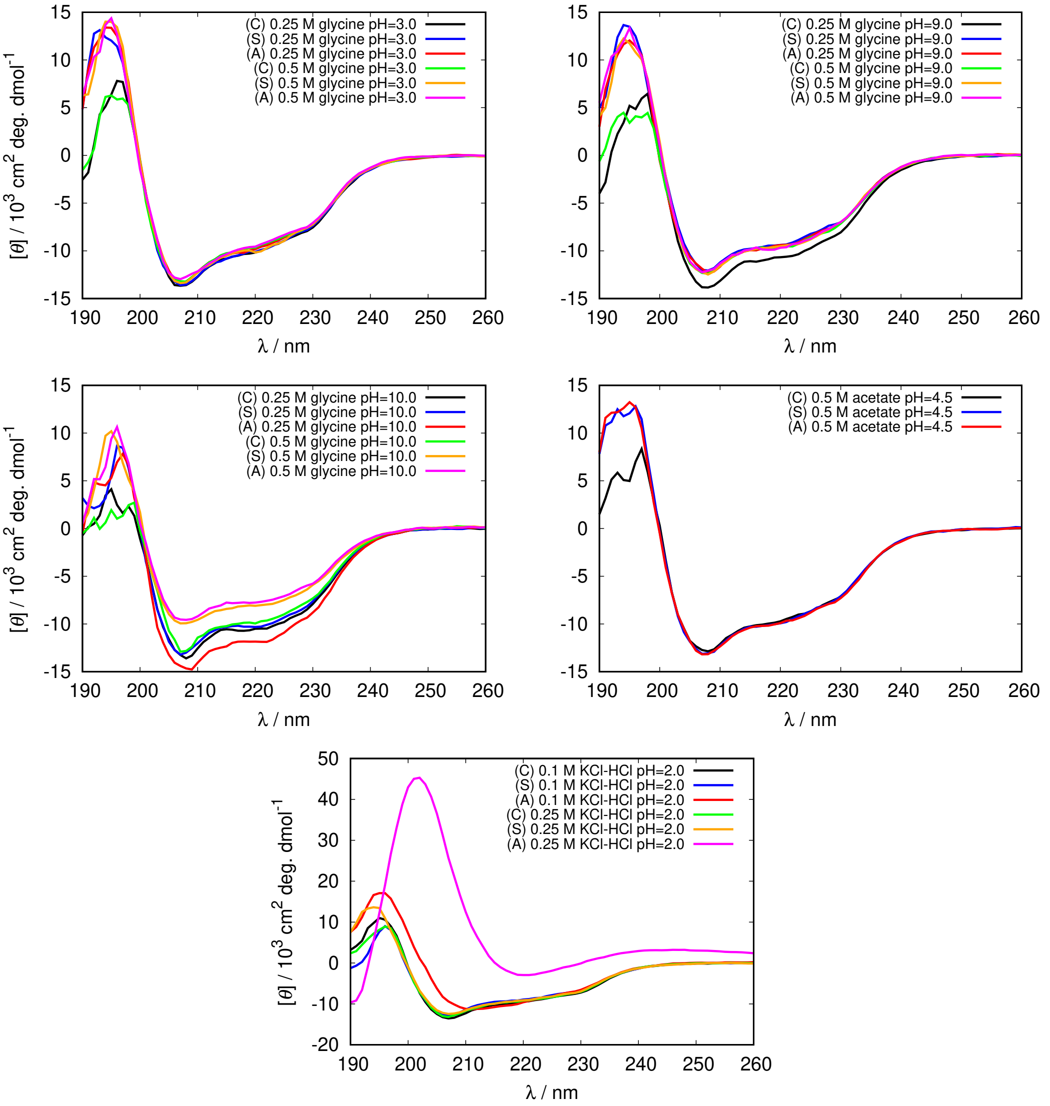

Figure S5. CD spectra of control (C), static (S) and agitated (A) HEWL in different buffer solutions. These results indicate that no fibrils were formed in any of the buffer solutions presented here, except for the agitated HEWL in 0.25 M KCl-HCl, where we can observe a distinct strong positive band at approximately 200 nm and a single negative minimum at approximately 220 nm, denoting the increased amount of β-structure in this sample. The agitated and static samples in 0.5 M glycine at pH=10.0 display only a minor decrease in α-helix content. Meanwhile, the CD spectra for agitated HEWL in 0.1 M KCl-HCl buffer solution shows a slightly deviating curve from the control sample, but without a higher amount of β-structure present.

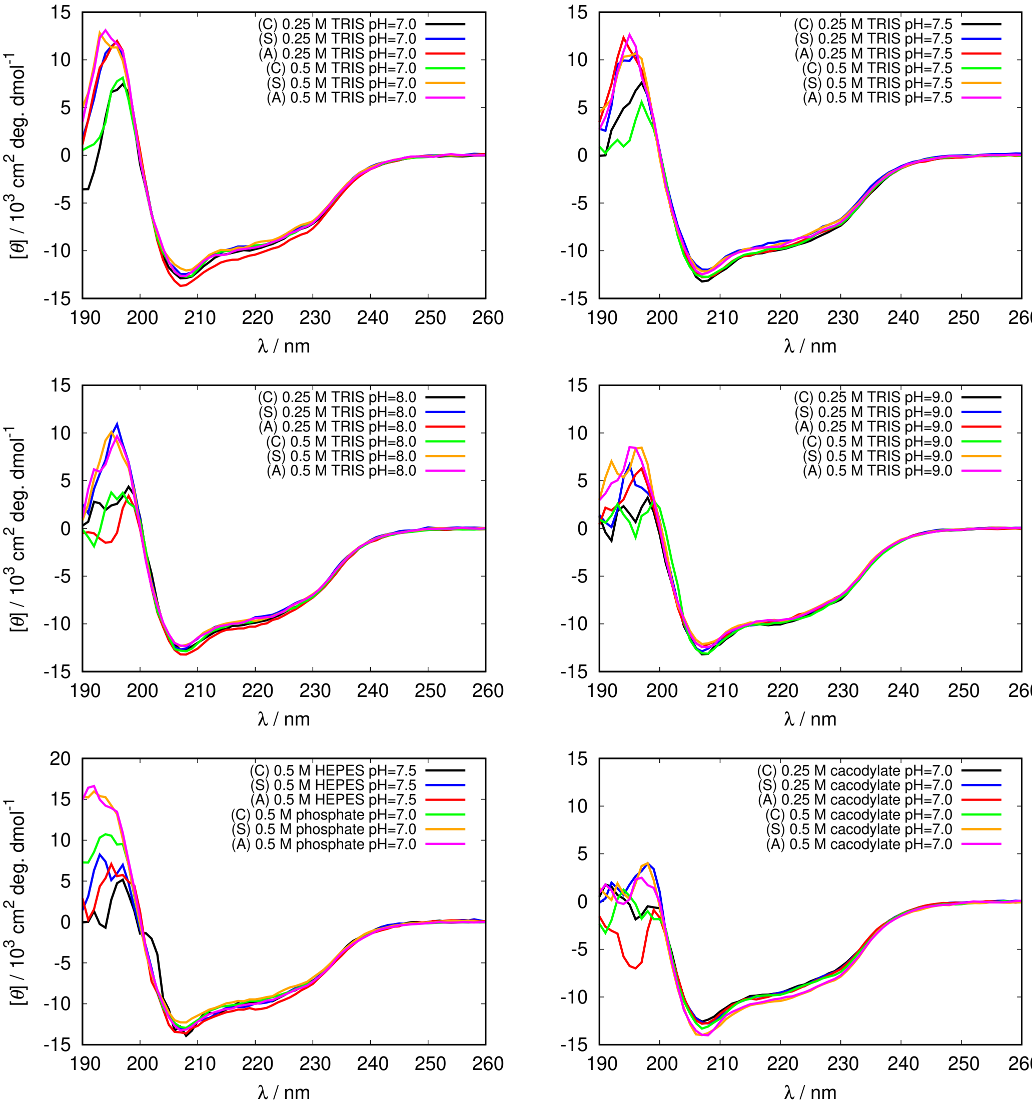

Figure S6. CD spectra of control (C), static (S) and agitated (A) HEWL in different buffer solutions. The CD spectra of TRIS, cacodylate, HEPES and phosphate buffer solutions presented here show no decisive alterations among samples with diverse incubation. Thus no fibrils were formed in any of present HEWL solutions.

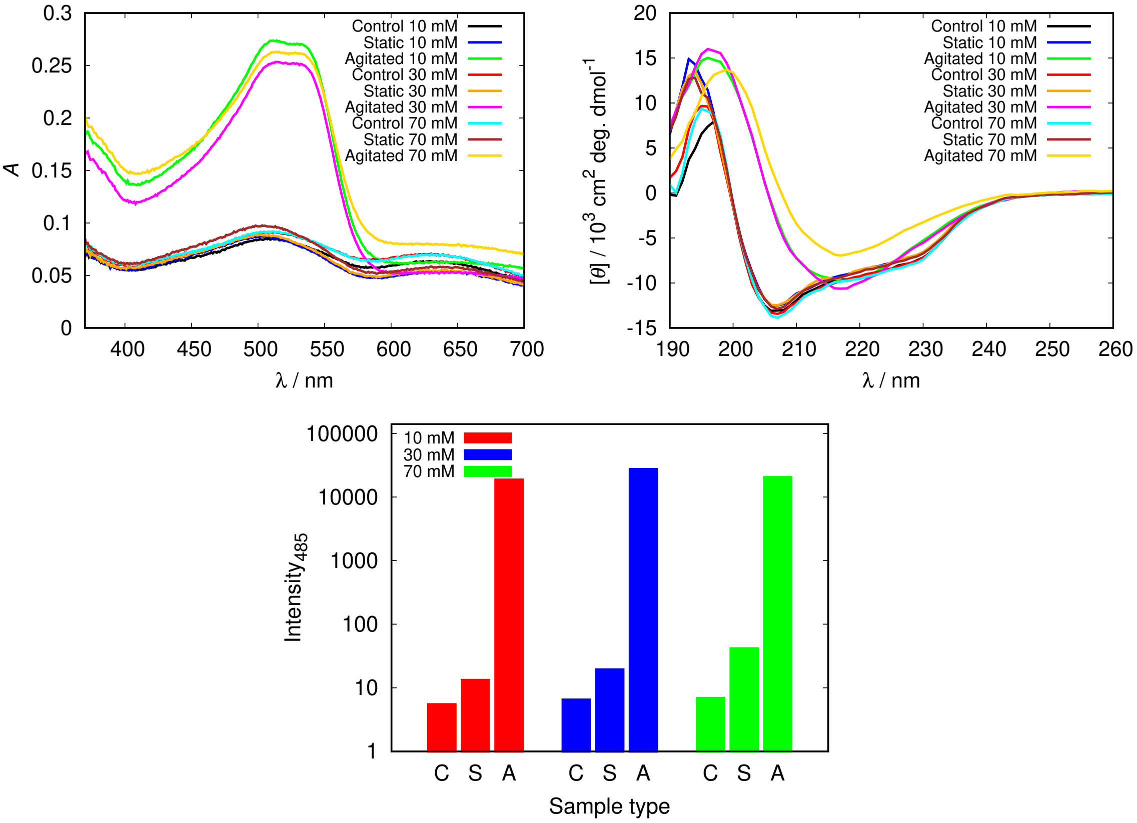

Figure S7. Congo red absorption spectra of HEWL and CD spectra of HEWL (top) and the intensity of ThT fluorescence emission at 485 nm for control (C), static (S) and agitated (A) samples of HEWL (bottom) in 0.25 M glycine at pH=2.0 with different concentrations of added NaCl. These results indicate fibrillization occurred in agitated HEWL samples at all concentrations of added NaCl.

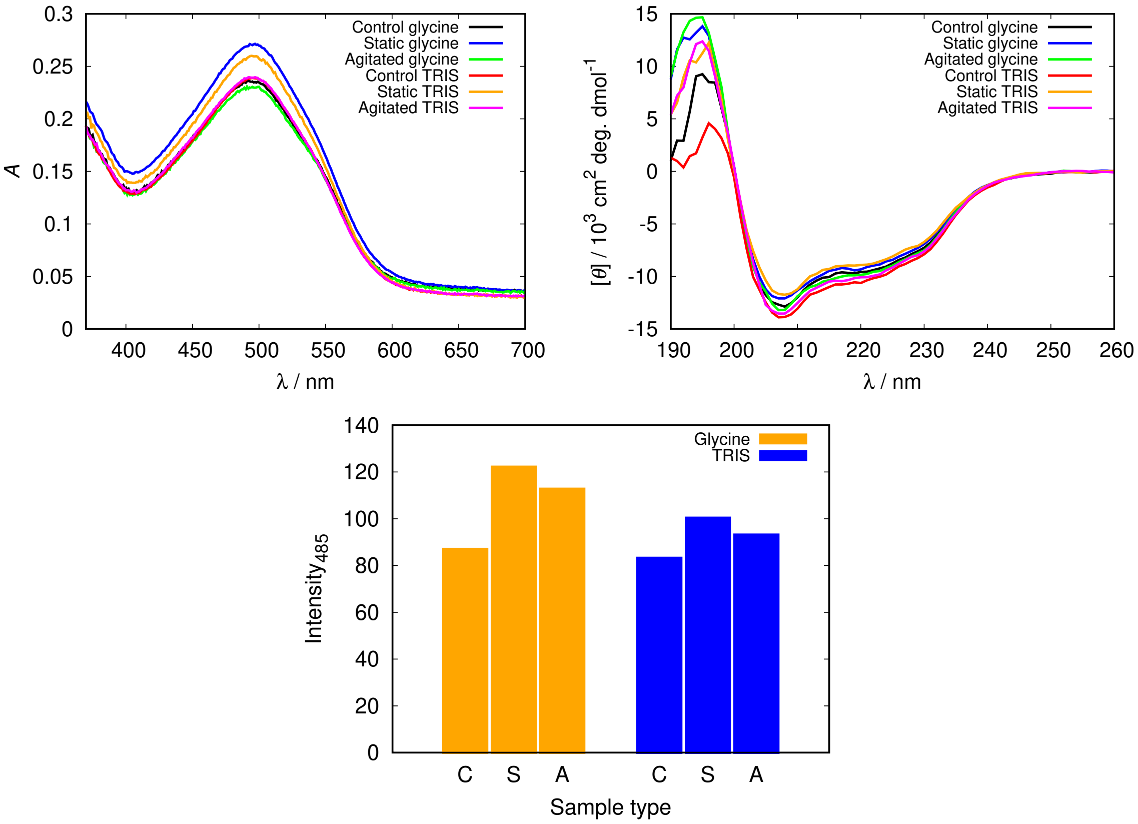

Figure S8. Congo red absorption spectra of HEWL and CD spectra of HEWL (top) and the intensity of ThT fluorescence emission at 485 nm for control (C), static (S) and agitated (A) samples of HEWL (bottom), compared in 0.25 M glycine and TRIS at pH=9.0 with both containing 50 mM NaCl. In the basic range of glycine buffer NaCl had no effect on the fibrillization of HEWL, as there are no meaningful differences between samples of varied incubation. Similar is the case for TRIS buffer solutions, in which NaCl could also not trigger fibril formation.

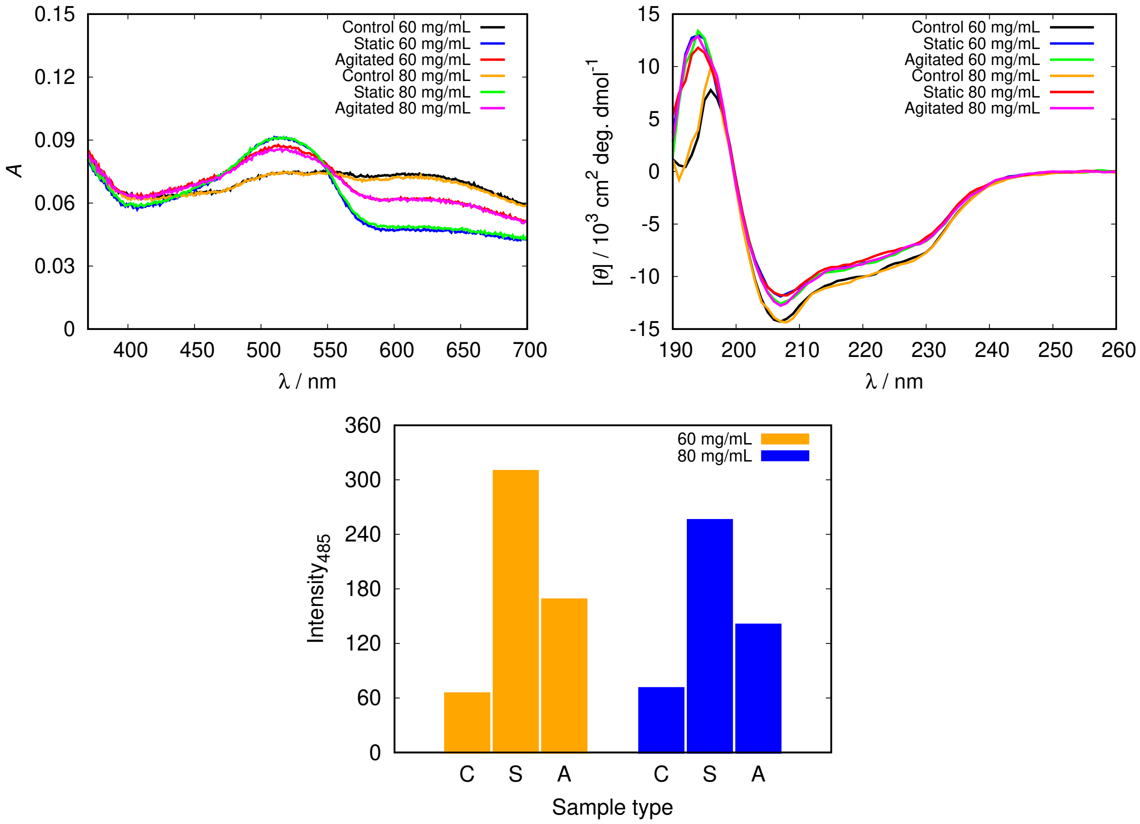

Figure S9. Congo red absorption spectra of HEWL and CD spectra of HEWL (top) and the intensity of ThT fluorescence emission at 485 nm for control (C), static (S) and agitated (A) samples of HEWL (bottom) in 0.5 M glycine at pH=2.0 with different concentrations of added PEG12000. It was already shown in the manuscript that 40 mg/mL of added PEG12000 is enough to prevent HEWL from fibrillizing. Here it is shown that by further increasing the concentration of PEG12000 the potential for fibrillization is gradually decreasing. The absorption spectra of Congo red and CD spectra of agitated and static samples hardly differ from control samples and the emission intensity of ThT for these samples is also decreasing with rising PEG12000 concentration.
